# Supplementary material for: Are Dietary Habits the Missing Link Between Hashimoto’s Thyroiditis and Osteoporosis?
Source: Nutrients. 2025 Jun 25;17(13):2109. doi: 10.3390/nu17132109 (PMC12252081; doi:10.3390/nu17132109)
Supplement: Supplementary file 1 [file nutrients-17-02109-s001.zip › nutrients-3686488-supplementary.pdf]

Table S1. Studies regarding Thyroid stimulating hormone and Thyroid hormone role on bone health

First name	Years	Study type	Main findings	Limits
**Murphy [19]**	2010	Ps	- 1278 healthy euthyroid postmenopausal women not receiving bone drugs were enrolled and followed for 6 years;<br>- Higher FT4 (β -0.091) and FT3 (β -0.087) were associated with reduced BMD;<br>- Higher FT4 was associated with increased bone loss at the hip (β -0.09);<br>- TSH was protective and the risk was reduced by 35%.	- Lack of previous medical history;<br>- Blood samples were from non-fasting subjects limiting comparability with other studies;<br>- Low number of hip fractures.
**Mazziotti [18]**	2010	Os	- 130 postmenopausal women were enrolled: 80 with osteoporosis, 49 with osteopenia and 1 with normal BMD;<br>- Low-normal TSH levels were associated with a high prevalence (35%) of vertebral fractures independently of THs, age and BMD.<br>- Women with higher FT4 (increased by 20%) and FT3 (increased by 33%) presented a higher risk of nonvertebral fractures and protection from nonvertebral fractures with higher TSH (reduced by 35%);<br>- Thyroid status within the upper normal range was associated with reduced BMD at the hip and an increased risk of nonvertebral fracture;<br>- Multivariate logistic regression analysis demonstrated that low serum TSH maintained a significant correlation with vertebral fractures even after correction for age (OR 1.1), low body mass index (OR 0.78), and BMD (OR 3.14).	- Study design;<br>- Reduced number of enrolled subjects.
**Leader [20]**	2014	Ps	- 14,322 subjects with a normal TSH were enrolled: 4269 with low-normal TSH, 3767 with intermediate TSH and 1385 with high-normal;<br>- Low TSH levels within the normal reference range are associated with a 28% increase in the risk for hip fractures in women only [OR 1.28, 95% CI (1.03-1.59)];<br>- The increased risk remained statistically significant after adjustments for age, relevant comorbidities, and bone drugs	- Single THS measurement.
**Grimnes [21]**	2008	Os	- 1961 subjects were enrolled: 993 postmenopausal women and 968 men;<br>- Subjects with low TSH levels presented lower BMD;<br>- Women with high serum TSH had significantly higher BMD;<br>- Within the normal TSH range, BMD was constant in both genders.	- Study design;<br>- Population selected using self-administered questionnaires;<br>- Blood samples were from non-fasting subjects.
**Segna [22]**	2017	MA	- 6 cohort study were included with a final sample comprised 5458 subjects (4723 with euthyroidism, 451 with subclinical hypothyroidism and 284 with subclinical hyperthyroidism);<br>- Median follow-up was 6.7 years;<br>- Subclinical hyperthyroidism was associated with a greater bone loss at the femoral neck compared to euthyroid subjects (-0.18 %ΔBMD);	- Study design;<br>- No uniformity of collected data;<br>- Poor generalizability of data;<br>- Limited power of analysis shown by large confidence intervals.

			- Subjects with THS lower that 0.10 mUI/L showed a double to 3-fold annualized rate of hip bone loss;<br>- Subclinical hypothyroidism was not associated with bone loss compared to subjects with euthyroidism.	
**Bauer [23]**	2001	Ps	- 9704 postmenopausal women were enrolled and followed for about 6 years;<br>- 332 women had a first hip fracture, 389 an incident vertebral fracture and 2520 nonvertebral fractures;<br>- Women with TSH levels of 0.1 mIU/L or less had a significantly increased risk of new hip [relative hazard 3.6; 95% CI (1.0, 12.9)] and vertebral [OR 4.5, 95% CI (1.3, 15.6)] fractures], independently of age, hyperthyroidism, self-rated health, and use of estrogen and thyroid hormone, compare with subjects with TSH levels in the normal range (0.5-5.5 mU/L);<br>- The risk of vertebral fracture, but not hip or other nonvertebral fractures, was significantly elevated among women with borderline low TSH levels (0.1 to 0.5 mIU/L);<br>- Women with previous hyperthyroidism had an increased risk for hip fracture, but no evidence that thyroid hormone use itself was associated with an increased risk of fracture.	
**Abrahamsen [24]**	2014	Os	- 6629 women and 2588 men with low TSH and 122,400 women and 99,738 men with normal THS were enrolled;<br>- During a median follow-up of 7.5 years, 16,543 subjects had at least one major fracture;<br>- Incidence rates for hip fractures were significantly higher in patients with low TSH;<br>- In subjects with normal TSH the risk of major osteoporotic fractures increased for each 1 standard deviation that TSH was below the mean. For hip fractures, the multiply adjusted hazard ratio per standard deviation was 1.45.<br>- The risk of hip fractures was significantly higher with lower euthyroid TSH levels in both genders, while the association with major osteoporotic fractures was observed in women only;<br>- Overt thyrotoxicosis was significantly associated with an increased risk of hip fracture in the multiply adjusted analysis<br>- The risk of major osteoporotic fractures was increased significantly in subclinical thyrotoxicosis but not in overt thyrotoxicosis.	- Study design;<br>- Hospital setting resulted in higher fracture rates compared general practice.
**Blum [25]**	2015	MA	- 13 prospective cohort study were collected with a final sample consisted of 70,298 subjects;<br>- 63,987 subjects were euthyroid, 4092 had a subclinical hypothyroidism and 2219 a subclinical hyperthyroidism, 1669 low TSH (0.10–0.44 mIU/L) and 550 suppressed TSH;	- Study design;<br>- No uniformity of data collected;<br>- Lack of relevant data with a possible residual counfunding.

			- Subclinical hyperthyroidism was associated with an increased risk for hip and other fractures. In age- and sex-adjusted analyses, the hazard ratio for subclinical hyperthyroidism vs euthyroidism was 1.36 for hip fracture, for any fracture, hazard ratio was 1.28, for nonvertebral fracture, hazard ratio was 1.16, and for vertebral fracture was 1.51;<br>- The highest risks of fractures were observed in individuals with suppressed TSH or with subclinical hyperthyroidism. Hazard ratio was 1.61 for hip fracture, for any fracture was 1.98, for nonvertebral fracture, was 1.61, and for vertebral fracture, was 3.57.<br>- No association between subclinical hypothyroidism and fractures was found;	
**Van Der Deure [37]**	2008	Os	- 4801 subjects were enrolled and 1151; only for 1151 subjects thyroid parameters were available;<br>- Femoral neck BMD and cortical thickness increased with serum TSH (β 0.009). The result was not confirmed after adjusting for age, gender and BMI;<br>- FT4 was negatively associated with bone parameters, also after adjusting for age, gender and body mass index: lumbar spine BMD β -0.003, femoral neck BMD β -0.005, and cortical thickness β -0.001;<br>- The TSHR-Glu^{727} allele was associated with a higher BMD at the femoral neck and lower serum TSH;<br>- No association of the TSHR polymorphism or serum TSH or FT4 with fracture risk was found.	- Study design.
**Liu [38]**	2025	MR	- Both hyperthyroidism (OR 1.08) and hypothyroidism (OR 1.183) showed a notable association with osteoporosis;<br>- TSH was a mediator in the causal association between hypothyroidism and osteoporosis;<br>- FT4 levels due to hyperthyroidism increased the risk of osteoporosis.	- MR analyses were conducted exclusively in European populations;<br>- The biological functions of some SNP-associated genes are still unclear, potentially affecting result interpretation.
**Leng [39]**	2024	MR	- Hypothyroidism and hyperthyroidism acted as risk factors for osteoporosis in two-sample MR analyses;<br>- After adjusting for confounders, including diabetes mellitus, hyperthyroidism was no longer a significant risk factor;<br>- Hypothyroidism may elevate the risk of osteoporosis (OR 1.082) by altering blood metabolite levels, such as triglycerides.	- MR analyses were conducted exclusively in European populations;<br>- Diabetes mellitus was the only intervening variable analyzed.

Ps: prospective study; Os: observational study; MA: meta-analysis; MR: Mendelian randomization; BMD: bone mineral density; THs: thyroid hormones; FT3: free tri-iodothyronine; FT4: free thyroxine; TSH: thyroid stimulating hormone.

Table S2. Studies regarding the role of determinants of autoimmune thyroid disease on bone health

First name	Years	Study type	Main findings	Limits
**Deng [29]**	2021	Os	- 114 men were enrolled and divided into two groups: 39 with low-level serum TSH group (TSH ≤ 1.65 mU/L) and 75 with high-level serum TSH group (TSH > 1.65 mU/L);<br>- Low BMD is related to higher-than-normal levels of FT3 and FT4;<br>- TSH serum concentrations positively correlated with BMD in men with normal thyroid function;<br>TSH can promote osteoblast proliferation and increased the expression of osteoblast differentiation genes (ALP, BMP2, COL1, and Runx2) in a dose-dependent manner.	- Study design;<br>Limited sample size.
**Lambrinoudaki [44]**	2017	Os	- 335 postmenopausal women were enrolled;<br>- Women with prevalent vertebral fractures exhibited lower levels of FT3 compared to women without vertebral fractures (2.27 ± 0.48 vs 2.60 ± 0.60 pg/mL);<br>- Higher TSH levels within the normal range were associated with lower risk of vertebral fracture;<br>- Women with positive TPOAb and TgAb presented the highest risk of vertebral fracture compared to women with only one positive category of antibodies or women without antibodies (26.3 % vs 5.3% and 5.9%, respectively);<br>- Positivity for TgAb and both TPOAb and TgAb increased risk of vertebral fractures (OR 3.308 and 6.328, respectively);<br>- The results were confirmed independently of age, menopausal age and body mass index.	- Study design;<br>- Non-generalizability of results;<br>- No bone turnover markers available.
**Polovina [45]**	2017	Os	- 189 postmenopausal women were enrolled: 110 euthyroid and 79 with subclinical hypothyroidism;<br>- The enrolled women were divided into two different groups according to the positivity of TPOAb;<br>- Euthyroid subjects with autoimmune thyroiditis had lower BMD at the spine and hip, and a higher prevalence of fractures;<br>- TPOAb were found to be strongly linked with increased risk of any type of fractures, independently of TSH values (OR 7.8).	- Study design;<br>- Lack of data regarding<br>- duration of autoimmune thyroid disease;<br>- No information regarding the time of occurrence of fracture.
**Polovina [46]**	2013	Os	- 2 postmenopausal women with newly discovered subclinical hypothyroidism and 51 matched controls were enrolled;<br>- FRAX score calculation and measurement of bone markers<br>- were performed;<br>- The FRAX score was significantly higher in the group with subclinical hypothyroidism than in the controls (6.5 vs 4.35);<br>- No significant difference in bone markers was observed.	- Study design.

**Chen [48]**	2020	MA	- 9 study were selected with a total of 1891 osteoporotic patients and 2027 healthy controls;<br>- 8 studies evaluated the association between IL-6 174G/C polymorphism and osteoporosis and 3 studies related to IL-6 572C/G (rs1800796);<br>- A higher osteoporosis risk was observed in IL-6 572C/G additive (OR 2.25), dominant (OR 1.42) and recessive (OR 1.96) model and a reduced osteoporosis risk in IL-6 572C/G C allele subjects.	- Limited sample size and gene polymorphism evaluated.
**Bhadricha [51]**	2021	Os	- 255 healthy participants were selected for the study, of which 140 premenopausal women were in the age group of 25–40 years and 115 postmenopausal women were in the age group of 50–65 years;<br>- Osteoporosis is more prevalent in postmenopausal women as bone loss is accelerated due to declining levels of estrogen;<br>- Postmenopausal women with normal BMD had elevated levels of Th17 cells compared to premenopausal women;<br>- A reduction in expression of CD25 in postmenopausal women compared to premenopausal women was observed;<br>- Th17/Treg was higher in postmenopausal women with normal as well as low BMD compared to premenopausal women.	- Study design;<br>- Limited sample size.
**Gerenova [54]**	2019	Os	- 85 women and 8 men with Hashimoto's Thyroiditis and 30 healthy controls were enrolled;<br>- Patients were divided into three subgroups: 33 with normal thyroid function, 11 with hypothyroidism and 49 with hypothyroidism;<br>- IL-23 (38.70 vs 14.76 pg/mL) and IL-17 (6.26 vs. 2.11 pg/mL) in Hashimoto's Thyroiditis patients were significantly different compare controls;<br>- The IL-17 and IL-23 levels correlated positively in the group of euthyroid patients (0.484), but not in other two groups;<br>- TPOAb were not related to IL17 and IL23.	- Study design;<br>- Limited sample size.

Os: observational study; MA: meta-analysis; Ps: prospective study; MR: Mendelian randomization; BMD: bone mass density; THs: thyroid hormones; FT3: free tri-iodothyronine; FT4: free thyroxine; TSH: thyroid stimulating hormone.

Table S3. Studies regarding the role oxidative stress on bone and thyroid health

First name	Years	Study type	Main findings	Limits
**Zhao [67]**	**2021**	MA	- 36 studies were included in the analysis with 5273 subjects (2088 postmenopausal women and 3185 healthy postmenopausal women);<br>- No significant difference was found in total oxidant state between osteoporotic women and controls;<br>- Both total antioxidant power was significantly decreased in the osteoporotic women compared with the control group [standardized mean differences (SMD) = − 3.18; 95% CI, − 5.21, − 1.15; *P* = 0.002; I^2 = 97.7%; TAC: SMD = − 2.56; 95% CI, − 4.41, − 0.70; *P* = 0.007; I^2 = 97.1%)];<br>- A significant decrease in catalise activity was found in osteoporotic patients compared with controls (SMD = − 1.95; 95% CI, − 3.30, − 0.60; *P* = 0.005; I^2 = 95.7%);<br>- Glutathione peroxidase activity was significantly lower in osteoporotic subjects than in controls (SMD = − 2.38; 95% CI, − 3.92, − 0.84; *P* = 0.002; I^2 = 96.5%);<br>- Result showed a significantly higher serum malondialdehyde level in the osteoporotic group compared with control group (SMD = 1.02; 95% CI, 0.35, 1.68; *P* = 0.003; I^2 = 94.1%);<br>- The level of advanced oxidation protein products showed to be significantly higher in the osteoporotic group than in the control group (SMD = 0.63; 95% CI, 0.10, 1.16; *P* = 0.019; I^2 = 79.4%);<br>- a significantly lower level of uric acid was found in the osteoporotic group compared to the control group (SMD = − 1.18; 95% CI, − 2.26, − 0.10; *P* = 0.033; I^2 = 97.2%)	- heterogeneity of selected studies;<br>- discrepancy due to different laboratory methods;<br>- many studies included Asian population.
**Ruggeri [75]**	2016	Os	- 134 subjects were enrolled: 71 with Hashimoto's Thyroiditis in euthyroidism and 63 healthy subjects;<br>- Reactive Oxygen Metabolites were significantly higher in Hashimoto's Thyroiditis patients compared to healthy controls and the two parameters were inversely correlated (-0.211)<br>- Biological Antioxidant Potential were lower in cases compared controls;<br>- In cases, the balance between antioxidants and oxidants was shifted in favor of the latter.	- Study design;<br>- Limited sample size.
**Rostami [76]**	2012	Os	- 44 subjects with Hashimoto's Thyroiditis and 58 healthy controls were enrolled;<br>- Cases showed higher levels of TSH (19.68 vs 1.4 mIU/L), TPOAb (475.07 vs 4.60), TgAb (653.62 vs 19.77) and larger thyroid volume than controls;<br>- Glutathione levels were higher in controls compared to Hashimoto's Thyroiditis (2.4 vs 6.2 mcml/L);<br>- Glutathione peroxidase was higher in Hashimoto's Thyroiditis compared to controls (329.5 vs 276.5 IU/L);<br>- Glutathione levels were inversely related to TPOAb (-0.326).	- Study design;<br>- Limited sample size.
**Ates [77]**	2015	Os	- 124 subjects were included: 93 with Hashimoto's Thyroiditis (31 with euthyroidism, 31 with subclinical hypothyroidism and 31 with overt hypothyroidism) and 31 healthy controls;<br>- Oxidative stress levels were higher in the overt hypothyroidism group;<br>- TPOAb were inversely related to total antioxidant agents (-0.282, in all population, -0.216 in euthyroidism, -0.245 in subclinical hypothyroidism and -0.589 in overt hypothyroidism).	- Study design;<br>- Limited sample size.
**Riis [81]**	2023	Ps	- 51 women with newly diagnosed of autoimmune thyroiditis and 18 controls were included in the analysis;	- Small sample size;<br>- Limited follow-up.

	- Twenty-two participants were diagnosed with mild (subclinical) hypothyroidism, and 23 with overt hypothyroidism;<br>- The mean follow-up was 442 days;<br>- The geometric mean of the excretion of 8-oxoGuo was 1.63 nmol/mmol creatinine at baseline and 1.67 nmol/mmol at euthyroidism (*P*= 0.39), while that of 8-oxodG was 1.28 nmol/mmol at baseline and 1.32 nmol/mmol at euthyroidism, respectively (*P* = 0.47);<br>- At the end of the follow-up; 25 patients exhibited an increase, while 20 patients showed a decrease in 8-oxoGuo;<br>- The geometric mean of the urinary excretion of 8-oxoGuo and 8-oxodG in the control subjects was 1.23 (95% CI: 1.07–1.42) and 1.04 (95% CI: 0.88–1.23) nmol/mmol creatinine, respectively. Significantly higher excretions of 8-oxoGuo (*P* < 0.001) and 8-oxodG (*P* = 0.03) were found in the patients at euthyroidism compared to the control subjects;<br>- A negative correlation between 8-oxoGuo and baseline TSH and positive correlations between 8-oxoGuo and baseline FT4 were found.

MA: meta-analysis; Os: observational study; Ps: prospective study; THs: thyroid hormones; FT3: free tri-iodothyronine; FT4: free thyroxine; TSH: thyroid stimulating hormone.

Table S4. Studies regarding the impact of dietary habits and vitamin D on bone and thyroid health

First name	Years	Study type	Main findings	Limits
**Wu [43]**	2024	Os	- 3865 study subjects were included in this analysis, 224 participants were TgAb positive and 356 were TPOAb positive. 392 participants reported a history of hip, spine or wrist fracture;<br>- There was a weak negative correlation between thyroid autoantibodies and the bone mineral density of the total femur (with TOPAb -0.047 and with TgAb -0.039), femoral neck (with TOPAb -0.052 and with TgAb -0.041), and total spine with (with TOPAb -0.030 and with TgAb -0.050);<br>- The median BMD values of the total femur, femoral neck and total spine were all significantly lower in participants with TgAb positive, as well as in participants with TPOAb positive;<br>- Thyroid-specific autoantibodies between males and females were significantly different (TgAb: 4.1% vs 7.6% and TPOAb:9.2% vs 12.75%);<br>- In women, age [OR 1.63; 95% CI (1.16; 2.28)], presence of TgAb and TPOAb [OR 2.3; 95% CI (1.25; 4.31)] and smoke [OR 1.93; 95% CI (1.38; 2.71)] increased risk of major fractures;<br>In men, alcohol history [OR 1.53; 95% CI (1.05; 2.24)], smoke [OR 1.49; 95% CI (1.13; 1.97)] and vitamin D levels [OR 1.84; 95% CI (1.34; 2.54)] increased risk of major fractures.	- Study design;<br>Use of self-reported questionnaires.
**Aleksandrova [85]**	2021	SR	- 29 articles (16 Os and 13 RCT) were included in the SR;<br>- The SR indicated an inverse relationship between plant-based diets and oxidative stress;<br>- The western diet was directly associated with oxidative stress and inflammation.	- Study design;<br>- Heterogeneity of data collected.
**Wu [89]**	2022	Ps	- 1254 subjects were enrolled and followed for 6 years;<br>- Subjects with low selenium levels and without selenium supplementation showed higher TPOAb levels and a higher rate of Hashimotos's Thyroiditis [relative risk 3.65; 95% C (1.03-12.90).	- No RCT with a placebo group.
**Wolf [92]**	2005	Os	- 11 068 postmenopausal women were enrolled;<br>- Dietary intake and serum levels of antioxidants were not associated with bone mineral density at the lumbar spine, total hip and total body;<br>- Vitamin C levels were related to the use of hormone therapy. The beneficial effect of hormone therapy uses on femoral neck BMD appears to be greater among women with higher total intakes of vitamin C (the group with a high current use compared to never or former user group had a difference of 0.42 g/cm^2 of bone mineral density)	- Study design;<br>- Use of self-reported food frequency questionnaire.
**Xue [93]**	2022	Os	- 21939 subjects were included;	- Study design;

			- The highest consumption of selenium from diet were associated with higher bone mineral density (total femur, neck, trochanter, intertrochanter and lumbar spine) compared to the lowest consumption (β=0.014, β=0.010, β=0.011, β=0.017 and β=0.013, respectively);<br>- The dose-response relationship showed an inverted U-shape relationship between dietary selenium intake and bone mineral density levels.	- Use of 24-hour dietary recall interview.
**Zhou [94]**	2024	Os	- 5618 participants were included;<br>- Higher dietary vitamin A, vitamin C, vitamin E, zinc, selenium, and total carotenoid, were positively associated with bone mineral density;<br>- Subjects with the highest levels of vitamin E, zinc and selenium had a low risk of osteoporosis [OR 0.65, 95% CI (0.46, 0.92); OR 0.581, 95% CI (0.408-0.826); OR 0.673, 95% CI (0.503-0.899), respectively];<br>- A composite dietary antioxidant index was used to evaluate the combined exposure of dietary antioxidant intake. Subjects with the highest quartile of composite dietary antioxidant index had a high bone mineral density at all evaluated sites;<br>- The association between the composite dietary antioxidant index and bone mineral density levels were more significant in women.	- Study design.
**Yu [100]**	2017	RCT	- 60 subjects with Hashimoto's Thyroiditis were enrolled and divided into two groups: 24 treated with levothyroxine (group 1) and 36 treated with levothyroxine and selenium (group 2);<br>- After treatment TPOAb (23.63% vs 32.0%), TgAb (35.8 vs 14.2%) and interleukin-2 (159.3 vs 227.5 pg/mL) significantly decreased in group 2 compared group 1;<br>- Subjects treated with the combination therapy showed a slower disease progression than subjects treated with levothyroxine alone.	- No double-blind design;<br>- Limited sample size
**Krysiak [101]**	2011	RCT	- The included patients were randomized in a double-blind manner to receive: 42 treated with levothyroxine sodium, 43 with selenomethionine, 43 with levothyroxine sodium plus selenomethionine, and 42 with placebo;<br>- Levothyroxine and selenomethionine produced a similar systemic anti-inflammatory effect;<br>- The anti-inflammatory effect was strongest in subjects treated with levothyroxine sodium plus selenomethionine	- Limited sample size;<br>- Only women included.
**Wu [108]**	2015	Ps	- 118,085 subjects without a medical history of cardiovascular disease or cancer were enrolled. The mean follow-up was 25 years;<br>- The highest intake of whole grain was associated with a reduced risk of cardiovascular mortality [OR 0.85; 95%CI (0.78-0.92)];<br>- The associations between whole grain intake and cancer mortality were not significant.	
**Hodge [113]**	2007	Os	- 4439 subjects born in Australia, Greece or Italy were enrolled and evaluated through a validated food frequency questionnaire of 121 items;<br>- A correlation was found between intakes of monounsaturated fatty acids and polyunsaturated fatty acids;	- Study design.

			- Plasma phospholipid DHA and EPA increased with increasing intake of fish and were higher in people taking supplements of fish or cod-liver oil;<br>- Increasing intakes of dairy foods and butter were associated with higher plasma phospholipid percentages of conjugated linoleic acid and pentadecanoic acid;<br>- Plasma phospholipid oleic acid levels increased with increasing consumption of olive oil, and both linoleic acid and total trans fatty acids increased with increasing margarine intake	
**Esmaillzadeh [117]**	2007	Os	- 486 healthy women were enrolled and evaluated by using a 168-item food frequency questionnaire;<br>- 3 major dietary patterns were identified: healthy dietary pattern, Western dietary pattern and the traditional dietary patter;<br>- Compared to participants in the lowest quintile, those in the highest quintile of the healthy dietary pattern had significantly lower body mass index (25.7 vs 30.4 kg/m^2) and significantly lower prevalence of the metabolic syndrome (20% vs 37%);<br>- In comparison with participants in the lowest quintile, those in the highest quintile of the Western dietary pattern had significantly higher BMI (29.6 vs 26.3 kg/m^2), and had significantly higher prevalence of obesity (44% vs 23%) and the metabolic syndrome (39% vs 17&);<br>- Participants in the highest quintile of the traditional dietary pattern were significantly older (51 vs 45 years), slightly more physically active (15.6 vs 13.9 METh/wk), and significantly less likely to be obese than were those in the lowest quintile (31% vs 35%);<br>- Participants in the highest quintile of the healthy dietary pattern score had lower odds of the metabolic syndrome [OR: 0.55; 95% CI (0.27, 0.74)] and insulin resistance [OR:0.47; 95% CI (0.18, 0.89)] than did those in the lowest quintile, also after adjustment for the major confounding variables;<br>- Subjects in the highest quintile of the Western dietary pattern score had greater odds of the metabolic syndrome [OR: 1.73; 95% CI (1.11, 2.06)] and insulin resistance (1.33; 1.05, 1.84) than did those in the lowest quintile, also after adjustment for the major confounding variables.	- Study design;<br>- Only women enrolled.
**Henjum [122]**	2023	Os	- 205 subjects (115 vegans, 55 lacto-ovo vegetarians and 35 pescatarians) were enrolled;<br>- The prevalence of subclinical hypothyroidism did not differ between vegans, lacto-ovo vegetarians, and pescatarians. The results showed a low prevalence of thyroid dysfunction;<br>- In an unadjusted model, a significant association was found between vegan diet and thyroglobulin levels. Vegans had higher thyroglobulin levels compared to pescatarians [18 (10, 36) vs 11(5, 20) mcg/L];<br>- The mean concentrations of iodine in vegans, lacto-ovo vegetarians and pescatarians indicated milt-to moderate iodine deficiency [57 (28, 130) µg/L].	- Study design;<br>- Limited sample size.
**Chrysohoou [129]**	2004	Os	- 1514 men and 1528 women were randomly enrolled from Greece and divided according to tertile of diet score for the adherence to Mediterranean diet;	- Study design,

			- Subjects in the highest tertile of the score had lower prevalence of hypertension (20% vs 51% men, 10% vs 50% women); higher levels of serum HDL (49 vs 42 mg/dl men, 54 vs 49 mg/dl women); the multiple regression analysis confirmed the association between high diet score and low atherosclerotic marker also after adjusting for age, gender, smoking, physical activity, BMI, hypertension, diabetes, hypercholesterolemia and family history of cardiovascular disease;<br>- The fruit consumption, vegetables and moderate alcohol were inversely associated with inflammatory marker levels;<br>- The benefits of the Mediterranean diet on inflammation were significant even in high-risk groups of participants.	
**Lopez-Garcia [130]**	2004	Os	- 732 women from the Nurses' Health Study cohort were enrolled;<br>- The 2 main dietary patterns evaluated were the prudent and the western. The prudent pattern was characterized by higher intakes of vegetables, fruit, legumes, whole grains, fish, and poultry, whereas the Western pattern was characterized by higher intakes of red meat, processed meat, refined grains, sweets, desserts, French fries, and high-fat dairy products;<br>- Compared to the subjects in the lowest quintile of the prudent group, those in the higher quintiles were more physically active and smoked less (11.1 vs 15.4 METh/wk);<br>- The subjects in the higher quintiles of the prudent pattern had lower intakes of saturated fat (18 vs 21.7 g/d) and trans fatty acids (2.1 vs 3.1 g/d), but higher intakes of polyunsaturated fat (11.3 vs 10.1 g/d), folate (489.4 vs 344.6 mcg/d), and fiber (22.6 vs 13.8 mcg/d);<br>- The subjects in the higher quintiles of the Western pattern had higher intakes of saturated fat (21.9 vs 17.3 g/d) and trans fatty acids (3 vs 2 g/d) but lower intakes of folate (359.7 vs 478.8 mcg/d) and fiber (15.9 vs 21.0 g/d);<br>- C reactive protein and E-selectin had a negative association with increasing quintiles of prudent group, whereas they showed increasing trends with increasing quintiles of the Western group.	- Study design.<br>- .
**Mena [131]**	2009	RCT	- 112 older subjects with diabetes or more than 3 cardiovascular risk factors were enrolled and randomized to 3 dietary intervention groups: Mediaterranean diet with supplemental virgin olive oil (group 1), Mediterranean diet with supplemetal nuts (group 2) and lo-fat diet (group 3);<br>- After 3 months, in group 1 monocyte CD49d and CD40 expression were downregulated by 19% and 8%, respectively, in group 2 monocyte CD49d and CD40 expression were also decreased by 22% and 7%, respectively;<br>- After 3 months, sICAM-1 and interleukin-6 decreased in group 1 and 2 and increased in the low-fat-diet group, whereas sVCAM-1 and C Reactive Protein decreased only in the group 1;	- Limited sample size;<br>- Short follow-up;<br>- Poor compliance monitoring.

			- Systolic blood pressure decreased by 5.64 mmHg and 28.81 mmHg in group 1 and 2, respectively;<br>- Fasting glucose (-8.02 mg/dL) and HOMA (-0.86) index decreased in group 1;<br>- HDL increased by 5.18 mg/dL and 2.45 mg/dL in group 1 and 2, respectively.	
**Ülker [132]**	2023	Ps	- Hashimoto's Thyroiditis women were enrolled and randomly divided into four groups: 10 following the Medeterranean diet, 10 the Gluten-free diet; 10 the Mediterranean gluten-free diet, and 10 controls;<br>- TSH levels did not show a statistically significant difference across the groups before the intervention, while a significant difference was found across the groups after the intervention: TSH levels in the Gluten-free group were lower than controls (1.47 uIU/mL vs 2.56 uIU/mL);<br>- FT_3 hormone levels increased significantly in all intervention groups after the intervention, with the highest increase in the Mediterranean diet group (2.50 pg/mL before intervention vs 2.86 pg/mL after intervention);<br>- TPOAb and TgAb levels decreased in the groups after the intervention and decreased more in the Mediterranean diet (70.76 vs 68.95 IU/mL and 20.0 vs 13.74 IU/mL, respectively) and Mediterranean gluten-free diet groups (257.56 vs 140.20 IU/mL and 32.66 vs 6.31, respectively).	- Limited sample size;<br>- Short follow-up;<br>- Only women enrolled.
**Ruggeri [133]**	2021	Os	- 200 healthy subjects (173 women and 27 men) were enrolled;<br>- 81 subjects received a diagnosis of Hashimoto's Thyroiditis; the rest formed the control group;<br>- Hashimoto's Thyroiditis subjects had higher TSH (2.1 vs 1.8 mIU/L) and lower FT4 (10.7 vs 11.6 pml/L) values compared to controls;<br>- Advanced glycation end products were increased in Hashimoto's Thyroiditis subjects (154.68 vs 101.78 AU/gprot);<br>- Glutathione peroxidase (0.64 vs 0.65 U/mL), thioredoxin reductase (1.58 vs 2.08 U/mL), and total plasma antioxidant activity (1.59 vs 1.80 mM TE) were lower than in controls;<br>- A significant inverse correlation between advanced glycation end products and total plasma antioxidant activity in all participants (P = 0.018), as well as in Hashimoto's Thyroiditis subjects (P = 0.013), but not healthy controls (P = 0.747);<br>- Hashimoto's Thyroiditis subjects reported higher intake frequencies of animal foods (meat, P = 0.0001; fish, P = 0.0001; dairy products, P = 0.004) compared to controls;<br>- The number of subjects who reported consumption of >3 servings per week of nuts was significantly lower in HT subjects compared to controls (23% versus 55%; P = 0.0005);<br>- In the multivariable logistic regression model, adherence to the Mediterranean diet, as evaluated by PREDIMED score, was a significant predictive factor of TPOAb positivity [OR 0.19; 95% CI (0.07-0.05)]	- Limited sample size;<br>- Study design.
**Bozkurt [138]**	2013	Os	- 540 subjects were enrolled and divided into 3 groups: 180 euthyroid patients with Hashimoto's Thyroiditis (group 1), 180 sex-, age-, and BMI-matched euthyroid subjects	- Study design.

			with newly diagnosed Hashimoto's Thyroiditis (group 2), and 180 healthy controls (group 3);<br>- Group 1 had the lowest vitamin D levels (11.4 ± 5.2 ng/mL) compared to Group 2 (13.1 ± 5.9 ng/mL, P = .002) and to controls (15.4 ± 6.8 ng/mL, P<.001);<br>- A direct correlation was found between thyroid volume and vitamin D levels (0.145);<br>- A negative correlation was found between vitamin D levels and TPOAb (-0.361), and TgAb (-0.335).	
**Aktaş [139]**	2019	Os	- 130 Hashimoto's Thyroiditis patients were enrolled and divided into two groups: 60 with vitamin B12 levels below 200 pg/mL and 70 with vitamin B12 levels equal to or above 200 pg/mL;<br>- Patients were then divided into four groups according to vitamin D levels: 5 with vitamin D levels higher than 30 ng/mL, 9 with vitamin D levels between 20 and 30 mg/ml, 43 with vitamin D levels between 10-20 ng/ml and 73 with vitamin D levels lower than 10 ng/ml;<br>- The TPOAb levels were significantly higher in patients with low vitamin B12 levels (484.7±377.1 vs 292±389.4 IU/ml);<br>- A negative correlation between TPOAb and vitamin B12 was found in all groups (- 0.39).	- Study design;<br>- Limited sample size.
**Chaudhary [141]**	2016	Ps	- 102 subjects with autoimmune thyroid disorder were enrolled and randomized into two groups;<br>- Ninety-three percentage patients showed low vitamin D levels, and all included subjects had TPOAb title positive;<br>- The TPOAb title was higher in subjects with the lowest vitamin D levels (832.1±260.5 vs 632.42±231.08);<br>- A negative correlation was found between vitamin D levels and TPOAb title also after adjusting for age (r = −0.184; P = 0.068);<br>- At 3 months of follow-up, there was a significant fall in serum TPOAb title in patients treated with vitamin D supplementation compared to controls (387.0 vs 553.5).	- Short follow-up<br>- Limited sample size;<br>- Study design.
**Chahardoli [142]**	2019	RCT	- 42 women with Hashimoto's Thyroiditis were recruited. The participants were divided into 2 randomly allocated groups (50 000 IU of vitamin D weekly or placebo) and followed for 3 months;<br>- After supplementation, the serum levels of vitamin D and calcium increased significantly and the levels of TSH and TgAb decreased significantly in the vitamin D group compared to baseline;<br>- No significant reduction of TPOAb was found in vitamin D group compared to placebo.	- Limited sample size;<br>- Short follow-up
**Shin [143]**	2014	Ob	- 304 subjects were enrolled and divided into two groups: the autoimmune thyroiditis disorder group (111) and non-autoimmune thyroiditis disorder group (203);<br>- The prevalence of hyperthyroidism and hypothyroidism, and mean serum vitamin D levels in the patients with autoimmune thyroiditis disorders was significantly lower than in patients with non-autoimmune thyroiditis disorders (56.8% vs. 16.1%, 11.9% vs. 16.2%, 12.6±5.5 ng/mL vs. 14.5±7.3 ng/mL, respectively);	- Study design;<br>- Lack of data regarding agents that could impact

			- TPOAb and anti-TSH Receptor were also significantly higher in the autoimmune thyroiditis disorder group than in the non-autoimmune thyroiditis disorder group (298.5±336.7 IU/mL vs. 6.4±2.6 IU/mL, and 5.11±7.97 IU/L vs. 1.40±3.45 IU/L, respectively);<br>- In the autoimmune thyroiditis disorder group, the TPOAb level was inversely correlated with vitamin D (r=-0.252);<br>- High vitamin D levels decreased the TPOAb title [OR 0.92; 95% CI (0.86-0.95)], also after adjusting for age, gender, body mass index, goiter and diffuse thyroiditis.	thyroid function.
**Robat-Jazi [144]**	2022	RCT	- 40 subjects with Hashimoto's Thyroiditis were included and were randomly divided into two groups: 20 treated with vitamin D and 20 with placebo;<br>- After the intervention, the serum concentration of interferon-γ significantly decreased from 13.86±7.91 to 8.39±4.76 pg/mL in the vitamin D group and from 14.13±8.19 to 8.81±5.55 pg/mL in the placebo group;<br>- After supplementation, the serum concentration of Tumor Necrosis Factor-α was decreased significantly from 29.70±18.43 to 15.25±10.84 pg/mL in the vitamin D group and from 26.66±24.81 to 12.33±11.83 pg/mL in the placebo group;<br>- A negative correlation was found between low dose of levothyroxine and the serum level of TSH (-0.53);<br>- A correlation was found between the levels of TSH and interferon-γ (0.38).	- Limited sample size;<br>- Study design.
**Sarmiento-Ramón [145]**	2022	Os	- 60 patients affected by Hashimoto's Thyroiditis were enrolled;<br>- No relationship was found between vitamin D levels and thyroid antibodies.	- Study design;<br>- Limited sample size
**Kaan Demircioglu [146]**	2021	Os	- 256 subjects were included in this analysis and divided into two groups: 108 with Hashimoto's Thyroiditis and 148 without Hashimoto's Thyroiditis;<br>- Moreover, subjects were distributed in quartiles according to their preoperative vitamin D and TSH values (Vitamin D levels were determined as <6.21 ng/mL, 6.22–10.69 ng/mL, 10.7–22.68 ng/mL, and >22.69 ng/mL for the first, second, third, and fourth quartiles, respectively. TSH levels were determined as <0.94 uU/mL, 0.95–1.58 uU/mL, 1.59–2.4 uU/mL, and >2.4 uU/mL for the first, second, third and fourth quartiles, respectively);<br>- A positive correlation was found between Hashimoto's Thyroiditis and preoperative TSH (r=0.161, p=0.010), TPOAb (r=0.262, p=0.000), and preoperative TGAb (r=0.171, p=0.016) values, but negative correlation was found with age (r=–0.152, p=0.015);<br>- No positive significant correlation was found between Hashimoto's Thyroiditis and vitamin D level (r=0.104, p=0.095).	- Study design.
**Jiang [147]**	2022	MA	- 6 studies and 258 patients were included in the analysis;<br>- Combined results indicated vitamin D significantly reduced the level of TPOAb compared to the control group (WMD = −158.18, 95% CI: −301.92, −14.45, p = 0.031; I^2 = 68.8%, $p_{heterogeneity}$ = 0.007);<br>- Vitamin D did not significantly reduce the level of TG-Ab compared to the control group (WMD = −68.21, 95% CI: −143.04, 6.62, p = 0.074; I^2 = 35.8%, $p_{heterogeneity}$ = 0.183);	- Heterogeneity of data collected;<br>- Small samples size;

	- Combined results showed vitamin D did not significantly change the levels of TSH, FT3 and FT4 compared to the control group.	- Language limitation: only papers in English were included.

SR: systematic review; Ps: prospective study; Os: observational study; RCT: randomized controlled trial; MA: meta-analysis; FT3: free tri-iodothyronine; FT4: free thyroxine; TSH: thyroid stimulating hormone; TgAb: Thyroglobulin antibodies; TPOAb: Thyroid peroxidase antibodies; OR: odds ratio; CI: confidence interval
